# Protective role of exosomes in renal ischemia-reperfusion injury: a systematic review and meta-analysis

**DOI:** 10.3389/fphar.2025.1653907

**Published:** 2025-09-04

**Authors:** Weibo Wang, Supeng Tai, Xi Cheng, Lexing Yang, Yifan Chang, Junyi Yan, Junyue Tao, Jun Zhou

**Affiliations:** ^1^ Department of Urology, The First Affiliated Hospital of Anhui Medical University, Hefei, Anhui, China; ^2^ Institute of Urology, Anhui Medical University, Hefei, Anhui, China; ^3^ Anhui Province Key Laboratory of Genitourinary Diseases, Anhui Medical University, Hefei, Anhui, China

**Keywords:** renal ischemia-reperfusion injury, exosomes, mesenchymal stem cell, dose-response relationships, meta-analysis

## Abstract

**Introduction:**

Renal ischemia-reperfusion injury (RIRI) is a major cause of acute kidney injury (AKI), commonly triggered by clinical procedures such as nephrectomy, renal transplantation, or shock resuscitation, and may progress to chronic kidney disease (CKD). Although exosomes hold promise as nanotherapeutics with pleiotropic mechanisms for renal protection, robust preclinical validation remains limited. This study aimed to clarify the therapeutic potential of exosome-based interventions for RIRI and to explore factors that modulate their efficacy.

**Methods:**

This systematic review and meta-analysis synthesized data from 19 controlled preclinical studies involving 245 rodents, retrieved from the PubMed, Web of Science, Embase, and Cochrane Library databases, to evaluate the therapeutic efficacy of exosomes in experimental RIRI models.

**Results:**

Exosome treatment led to broad therapeutic improvements in renal function, renal damage, inflammation, oxidative stress, apoptosis, pyroptosis, cellular proliferation, and fibrosis. Subgroup analyses identified exosomal source as a critical determinant of efficacy, with mesenchymal stem cell- and endothelial colony-forming cell-derived exosomes outperforming those from fibroblasts. No clear dose-response relationship was observed, and while pre-treatment initially appeared more effective than post-treatment, this difference was not significant after adjusting for confounders. Notably, different administration routes yielded comparable therapeutic outcomes.

**Discussion:**

These findings underscore the renoprotective potential of exosome therapy in RIRI and highlight the need for further investigation to optimize therapeutic protocols and accelerate clinical translation.

**Systematic review registration:**

https://www.crd.york.ac.uk/PROSPERO/view/CRD420251008479, identifier PROSPERO, CRD420251008479.

## 1 Introduction

Renal ischemia-reperfusion injury (RIRI) refers to the pathophysiological process triggered by the restoration of renal blood flow after transient ischemia, leading to further tissue damage despite reperfusion (Grace, 1994). While reperfusion is essential for restoring renal viability, the abrupt reintroduction of oxygen paradoxically exacerbates cellular dysfunction through mechanisms such as uncontrolled oxidative stress propagation, dysregulated inflammatory cascades, and activation of programmed cell death pathways, ultimately resulting in secondary tissue injury (Eltzschig and Eckle, 2011). Moreover, progressive renal fibrosis secondary to unresolved IRI is both a pathological driver and a prognostic indicator of chronic kidney disease (CKD) development and progression (Zhang et al., 2024). Clinically, ischemia-reperfusion injury (IRI) represents a common complication in partial nephrectomy, renal transplantation, and shock management, contributing significantly to acute kidney injury (AKI) incidence and potentially to end-stage renal failure (Zuk and Bonventre, 2016). Although existing supportive care protocols and pharmacological interventions provide limited clinical benefits, the absence of definitive interventions highlights an urgent unmet need for innovative therapeutic strategies targeting RIRI pathogenesis.

Exosomes are 30–200 nm extracellular vesicles generated through the endosomal pathway, wherein intraluminal vesicles bud inward to form multivesicular bodies (MVBs) that subsequently fuse with the plasma membrane for cargo release (Pegtel and Gould, 2019). These nanoscale vesicular structures, ubiquitously distributed in biofluids, are enriched with molecular cargos such as regulatory proteins, coding/noncoding RNAs, and bioactive lipids. Exosomes facilitate intercellular communication and material exchange, exerting dual regulatory effects on tissue homeostasis maintenance and disease progression modulation (Kalluri and LeBleu, 2020). In chronic kidney disease (CKD) paradigms, exosome-based therapies have demonstrated preclinical and translational efficacy across multiple subtypes, including hypertensive, diabetic, and IgA nephropathy, as well as obstructive uropathy (Xiang et al., 2020; Martinez-Arroyo et al., 2021; Li H. et al., 2020; Song et al., 2024). Emerging evidence also supports their therapeutic potential in models of AKI (Li et al., 2024). Mechanistically, exosomes exert renoprotective efficacy *via* multipronged actions, including immunomodulatory effects, oxidative stress attenuation, cellular regenerative capacity enhancement, and fibrotic signaling suppression (Zhang et al., 2024). However, systematic validation of exosome-mediated renoprotection in RIRI, particularly regarding their mechanistic roles in mitigating reperfusion-induced secondary injury, remains limited. This study presents a systematic review and meta-analysis of preclinical studies to provide robust evidence supporting exosome-mediated renoprotection in RIRI. In addition, the analysis integrates functional outcome assessments with mechanistic insights and explores optimal strategies for exosome administration.

## 2 Materials and methods

### 2.1 Study design

This systematic review and meta-analysis adhered to Preferred Reporting Items for Systematic Reviews and Meta-Analyses (PRISMA 2020, Supplementary Table S1) guidelines and was prospectively registered with the International Prospective Register of Systematic Reviews (PROSPERO ID: CRD420251008479).

Two researchers systematically searched PubMed, Web of Science, Embase, and Cochrane Library databases from inception to 24 December 2024. Search strategies combined MeSH/Emtree terms and free-text keywords, targeting “kidney,” “ischemia-reperfusion injury” and “exosome.” The detailed search strategy is provided in Supplementary Table S2. Additionally, supplementary manual searches *via* Google Scholar and citation tracking of included studies were performed to minimize selection bias.

### 2.2 Inclusion and exclusion criteria

Eligibility followed the PICO (Population, Intervention, Comparison, Outcome) framework. The study population comprised rodent models (rats or mice) of RIRI. The intervention involved administering native exosomes, which are non-engineered vesicles naturally secreted by untreated mammalian cells, within RIRI models including bilateral renal ischemia, unilateral renal ischemia and unilateral ischemia combined with contralateral nephrectomy. Only experiments involving a single ischemia–reperfusion (I/R) procedure, with an ischemic duration between 20 and 60 min and a reperfusion period defined according to the time point of serum creatinine (SCr) measurement for the primary outcome, ranging from 24 h to 4 weeks, were included. The comparison was a control group receiving physiological saline, phosphate-buffered saline (PBS), or no treatment. The primary outcome was renal function, assessed by SCr levels. Secondary outcomes encompassed markers of kidney injury [blood urea nitrogen (BUN), kidney injury molecule-1 (Kim-1), kidney injury score], inflammation [interleukin-1 beta (IL-1β), tumor necrosis factor-alpha (TNF-α), neutrophil count], oxidative stress [catalase (CAT), malondialdehyde (MDA)], apoptosis [TUNEL-positive cells, Caspase-3, BCL-2-associated X protein (BAX), B-cell lymphoma 2 (BCL-2), phosphatase and tensin homolog (PTEN)], pyroptosis (Caspase-1), fibrosis [alpha-smooth muscle actin (α-SMA), fibrosis size], and cell proliferation (Ki67). Exclusion criteria encompassed non-rodent species, combination treatments, *in vitro* models, lack of controls, engineered or experimentally induced exosomes, and studies lacking statistical data required for meta-analysis of the primary outcome.

### 2.3 Data extraction

Citations from the systematic search were imported into EndNote (version X9.3.3), and duplicates were electronically removed. Records were initially screened by title and abstract, and full-text eligibility was assessed for retained studies. Two independent researchers applied inclusion and exclusion criteria, and eligible studies were selected. Discrepancies in study selection were adjudicated by a third reviewer.

Data extraction fields included: first author, publication year, country, animal model characteristics (species, strain, age, sex), animal sample size, I/R induction method, ischemia duration, exosome source, exosome dose, administration route, administration timing, and estimation time point. For studies lacking quantitative outcome data in text or table formats, corresponding authors were contacted *via* email to request raw datasets. Moreover, data extracted from figures were digitized using GetData Graph Digitizer (version 2.26). Two researchers independently performed data extraction, with discrepancies resolved through consultation with a third reviewer.

### 2.4 Study and evidence quality assessment

Methodological rigor of included animal studies was evaluated using the Systematic Review Center for Laboratory Animal Experimentation (SYRCLE)’s risk of bias tool (Hooijmans et al., 2014). Certainty of evidence for relevant outcomes was appraised with the GRADE (Grading of Recommendations Assessment, Development and Evaluation) framework (Guyatt et al., 2008). Disagreements in risk of bias or evidence grading were adjudicated by a third methodologist.

### 2.5 Statistical analysis

Given anticipated heterogeneity in outcome measurement methods, standardized mean differences (SMDs), calculated with Hedges’ g method which adjusted for small-sample bias, were selected to estimate effect sizes and their 95% confidence intervals (CIs). Meta-analyses were performed when at least two studies reported compatible outcomes, with summary effects visualized *via* forest plots and random-effects models applied *a priori* due to expected heterogeneity.

Heterogeneity was appraised through forest plot inspection, Chi-square (*χ* ^2^) tests, and quantified using the I-squared (*I* ^*2*^) statistic. Between-study variance was estimated using tau-squared (*τ* ^*2*^) to weight the random-effects model.

Preplanned subgroup analyses examined sources of heterogeneity (animal species, I/R model, exosome source/dose, ischemia duration, treatment timing, and evaluation time point), with SCr level as the predefined outcome indicator. Univariate meta-regression identified candidate factors influencing heterogeneity. Covariates with statistical significance (*P* < 0.10) in univariate analysis, along with potentially relevant but non-significant factors (*P* ≥ 0.10), were included in multivariable meta-regression models to assess their confounding effects on SCr outcomes. Nonlinear dose–response relationships between exosome dose and SCr reduction were modeled using restricted cubic spline (RCS) regression within a generalized linear modeling framework.

Sensitivity analysis was conducted to evaluate outlier-driven heterogeneity and assess the stability of the results. Each study was sequentially excluded, and the SMD was recalculated, with results displayed as a forest plot.

For outcomes with at least 10 studies, publication bias was assessed *via* Egger’s regression test and visual inspection of funnel plot symmetry. When asymmetry was detected, the trim-and-fill method was applied to adjust for bias, and adjusted funnel plots were presented to visualize corrected effect estimates. Adjusted pooled SMDs were reported to reflect potential bias-mitigated effects.

Analyses were conducted in R (version 4.4.1; R Foundation for Statistical Computing) using metafor package (version 4.6.0) for meta-analysis/regression and ggplot2 package (version 3.5.1) for visualization. Two-tailed *P* < 0.05 defined statistical significance.

## 3 Results

### 3.1 Study selection

**FIGURE 1:**
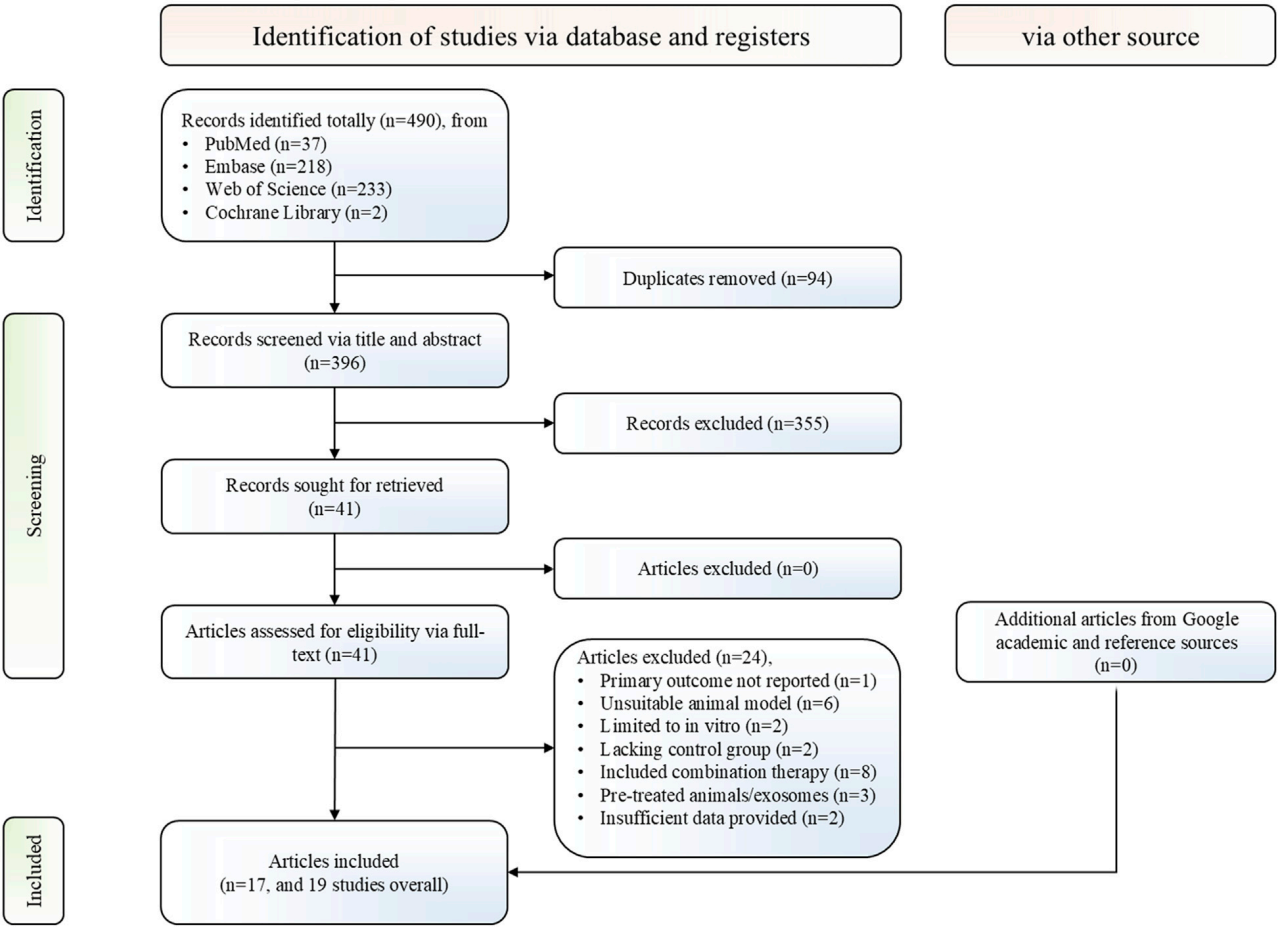
PRISMA flowchart for the study selection process. PRISMA, preferred reporting items for systematic reviews and meta-analyses.

A total of 396 non-duplicate records across four databases were retrieved based on the predefined search strategy. Following screening titles and abstracts, 355 records were excluded, and the remaining 41 articles underwent full-text review, yielding 19 controlled studies from 17 articles that met the predefined inclusion and exclusion criteria (Wang et al., 2014; Burger et al., 2015; Lin et al., 2016; Viñas et al., 2016; Vinas et al., 2018; Alzahrani, 2019; Li L. et al., 2019; Li et al., 2020b; Zhang et al., 2020a; Cao et al., 2021; Viñas et al., 2021; Xie et al., 2022; Liu and Shen, 2023; Wan et al., 2023; Abdelsalam et al., 2024; Liu et al., 2024; Yang et al., 2024). Furthermore, supplementary manual searches conducted *via* Google Scholar and citation tracking of included studies identified no additional eligible studies. A detailed PRISMA flowchart is presented in Figure 1.

### 3.2 Study characteristics

The analysis included 19 independent studies, involving 129 control animals and 116 treated animals. Among these studies, 11 utilized rat models and the remaining eight employed mouse models. The I/R models included unilateral renal ischemia, bilateral renal ischemia, and unilateral renal ischemia with contralateral nephrectomy, with ischemia durations ranging from 25 to 60 min. The therapeutic exosomes were derived from various sources, including stem cells, endothelial colony-forming cells, and fibroblasts, with doses ranging from 15 μg to 250 µg. Exosomes were administered *via* arterial, venous, or subcapsular routes in the kidney. Administration timings were categorized as pre-treatment or post-treatment. In all three pre-treatment studies, exosomes were administered 15 min prior to ischemia induction. Post-treatment timing varied, with exosomes administered from the onset of ischemia to up to 6 h post-reperfusion. Outcome assessments were performed at 24 h, 48 h, 72 h, and 4 weeks post-ischemia. The detailed study characteristics are summarized in Table 1.

**TABLE 1:** Characteristics summary of the included studies.

First author	Year	Country	Animals	Number of controls/treated	I/R model	Ischemic duration	Exosome source	Exosome dose	Administration route	Administration timing	Estimation time point	Reference
Wang	2014	China	SD rats (NR, 200–250 g)	3/6	Unilateral ischemia with contralateral nephrectomy	45 min	MSCs	100 ug	Carotid artery	Post-treatment	48 h	Wang et al. (2014)
Wang	2014	China	SD rats (NR, 200–250 g)	3/6	Unilateral ischemia with contralateral nephrectomy	45 min	Fibroblasts	100 ug	Carotid artery	Post-treatment	48 h	Wang et al. (2014)
Burger	2015	Canada	NOD-SCID mice (male, 6-8w)	6/7	Bilateral Renal Ischemia	30 min	ECFCs	15 ug	Jugular vein	Post-treatment	24 h	Burger et al. (2015)
Lin	2016	China	SD rats (male, 320–350 g)	8/8	Bilateral Renal Ischemia	60 min	ADMSCs	100 ug	Vein	Post-treatment	72 h	Lin et al. (2016)
Viñas	2016	Canada	FVB mice (male, 8–10w)	6/6	Bilateral Renal Ischemia	30 min	ECFCs	20 ug	Jugular vein	Post-treatment	24 h	Viñas et al. (2016)
Viñas	2018	Canada	FVB mice (male, 7–10w)	6/4	Bilateral Renal Ischemia	30 min	ECFCs	20 ug	Tail vein	Post-treatment	24 h	Vinas et al. (2018)
Alzahrani	2019	Saudi Arabia	NR. rats (female, 280–320 g)	5/5	Bilateral Renal Ischemia	45 min	MSCs	250 ug	Renal artery	Post-treatment	72 h	Alzahrani (2019)
Alzahrani	2019	Saudi Arabia	NR. rats (female, 280–320 g)	5/5	Bilateral Renal Ischemia	45 min	MSCs	250 ug	Renal artery	Post-treatment	4 w	Alzahrani (2019)
Li	2019	China	SD rats (male, 200–250 g)	6/6	Unilateral ischemia with contralateral nephrectomy	45 min	MSCs	30 ug	Carotid artery	Post-treatment	48 h	Li et al. (2019a)
Li	2020	China	SD rats (male, 200–250 g)	25/6	Unilateral ischemia with contralateral nephrectomy	45 min	HUSCs	20 ug	Right penile vein	Post-treatment	72 h	Li et al. (2020b)
Zhang	2020	China	SD rats (male, NR)	4/4	Unilateral Renal Ischemia	45 min	HUSCs	120 ug	Vein	Pre-treatment	24 h	Zhang et al. (2020a)
Cao	2021	China	C57BL/6 mice (male, 8–10w)	10/10	Bilateral Renal Ischemia	30 min	HucMSCs	200 ug	Vein	Post-treatment	48 h	Cao et al. (2021)
Viñas	2021	China	FVB mice (male, 7–10w)	4/5	Bilateral Renal Ischemia	30 min	ECFCs	20 ug	Tail vein	Post-treatment	24 h	Viñas et al. (2021)
Xie	2022	China	C57BL/6 mice (male, 6-8w)	3/3	Unilateral Renal Ischemia	25 min	BMSCs	100 ug	Vein	Post-treatment	72 h	Xie et al. (2022)
Liu	2023	China	C57BL/6 mice (male, 6-8w)	6/6	Unilateral Renal Ischemia	20 min	Fibroblasts	150 ug	Tail vein	Post-treatment	24 h	Liu and Shen (2023)
Wan	2023	China	SD rats (male,190–220 g)	3/3	Bilateral Renal Ischemia	40 min	HucMSCs	250 ug	Tail vein	Post-treatment	24 h	Wan et al. (2023)
Abdelsalam	2024	Egypt	SD rats (male, 211.36 ± 5.32 g)	8/8	Unilateral ischemia with contralateral nephrectomy	45 min	MSCs	150 ug	Subcapsular of the kidney	Pre-treatment	72 h	Abdelsalam et al. (2024)
Liu	2024	China	Balb/c mice (male, 10–12w)	12/12	Unilateral Renal Ischemia	30 min	MSCs	50 ug	Vein	Post-treatment	4 w	Liu et al. (2024)
Yang	2024	China	SD rats (NR, NR)	6/6	Unilateral Renal Ischemia	45 min	HUSCs	100 ug	Tail vein	Pre-treatment	24 h	Yang et al. (2024)

Abbreviations: NR, not reported; MSCs, mesenchymal stem cells; ECFCs, endothelial colony-forming cells; ADMSCs, adipose-derived mesenchymal stem cells; HUSCs, human urine-derived stem cells; HucMSCs, human umbilical cord mesenchymal stem cells.

### 3.3 Study quality assessment

Study quality was evaluated using the SYRCLE’s risk of bias tool, with detailed results summarized in Table 2. Random sequence generation was adequately implemented in only two studies. Although most studies reported comparable baseline animal characteristics, all failed to explicitly report allocation concealment procedures. Random housing during the experimental phases was described in 10 studies, while blinding protocols for caregivers or outcome assessors were not described in any study. No evidence was found for random selection process during outcome assessment across the studies. Incomplete outcome data, selective outcome reporting, or additional sources of bias were not identified in any included study.

**TABLE 2:** Risk of bias assessment of the included studies based on SYRCLE’s tool.

Study	Sequence generation	Baseline characteristics	Allocation concealment	Random housing	Blinding trial caregivers	Random outcome assessment	Blinding outcome assessors	Incomplete outcome data	Selective outcome reporting	Other sources of bias
Wang, 2014a	L	L	U	L	U	U	U	L	L	L
Wang, 2014b	L	L	U	L	U	U	U	L	L	L
Burger, 2015	U	L	U	U	U	U	U	L	L	L
Lin, 2016	U	L	U	U	U	U	U	L	L	L
Viñas, 2016	U	L	U	U	U	U	U	L	L	L
Viñas, 2018	U	L	U	U	U	U	U	L	L	L
Alzahrani, 2019a	U	L	U	L	U	U	U	L	L	L
Alzahrani, 2019b	U	L	U	L	U	U	U	L	L	L
Li, 2019	U	L	U	L	U	U	U	L	L	L
Li, 2020	U	L	U	L	U	U	U	L	L	L
Zhang, 2020	U	U	U	U	U	U	U	L	L	L
Cao, 2021	U	L	U	U	U	U	U	L	L	L
Viñas, 2021	U	L	U	U	U	U	U	L	L	L
Xie, 2022	U	L	U	U	U	U	U	L	L	L
Liu, 2023	U	L	U	L	U	U	U	L	L	L
Wan, 2023	U	L	U	L	U	U	U	L	L	L
Abdelsalam, 2024	U	L	U	L	U	U	U	L	L	L
Liu, 2024	U	L	U	L	U	U	U	L	L	L
Yang, 2024	U	U	U	U	U	U	U	L	L	L

Abbreviations: L, low risk of bias; H, high risk of bias; U, unclear risk of bias; SYRCLE, systematic review center for laboratory animal experimentation.

### 3.4 Meta analysis

#### 3.4.1 Primary outcome

**FIGURE 2:**
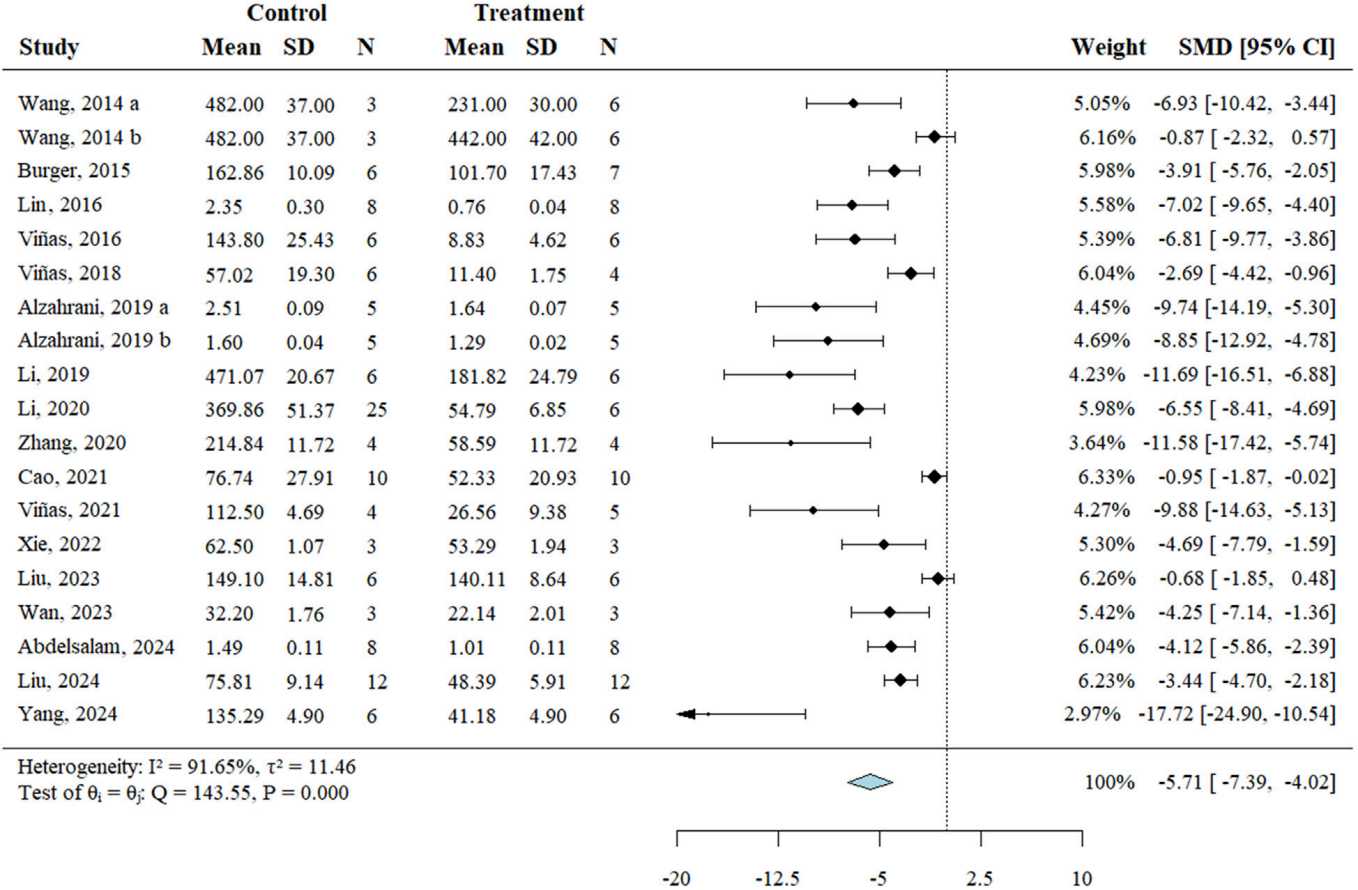
Forest plot of effects of exosome therapy on renal function in RIRI. RIRI, renal ischemia-reperfusion injury; SMD, standardized mean difference; CI, confidence interval; χ^2^, chi-square; I^2^, I-squared; τ^2^, tau-squared.

Renal function, assessed through SCr levels, served as the primary outcome. All 19 included studies reported changes in SCr and underwent systematic analysis to evaluate exosome therapy-induced renal functional improvements in RIRI. The meta-analysis demonstrated significantly lower SCr levels in exosome-treated animals *versus* controls (SMD = −5.71, 95% CI: −7.39 to −4.02, *P* < 0.001; *I* ^*2*^ = 91.60%) (Figure 2; Table 3). According to the GRADE framework, the evidence level was rated as high (Supplementary Table S3).

**TABLE 3:** Protective effects of exosomes in RIRI.

Outcome	Number of experiments	SMD [95% CI]	*P*	*I* ^*2*^ (%)	Level of evidence
Renal function					
SCr	19	−5.71 [−7.39, −4.02]	<0.001	91.60	High
Renal damage					
BUN	17	−4.78 [−6.49, −3.07]	<0.001	92.22	Moderate
Kim-1	2	−5.84 [−8.01, −3.66]	<0.001	47.98	Moderate
Kidney injury score	11	−4.96 [−6.68, −3.23]	<0.001	85.30	Moderate
Inflammation					
IL-1β	6	−3.57 [−8.13, 0.99]	0.125	97.70	Moderate
TNF-α	7	−3.84 [−6.43, −1.25]	0.004	92.92	Moderate
Neutrophil count	4	−7.35 [−9.12, −5.58]	<0.001	9.73	High
Oxidative stress					
CAT	3	12.06 [2.49, 21.63]	0.014	91.39	Moderate
MDA	3	−5.64 [−10.34, −0.95]	0.019	87.47	Moderate
Apoptosis					
TUNEL positive cell	9	−3.40 [−4.59, −2.22]	<0.001	70.78	Moderate
Caspase-3	9	−4.16 [−5.61, −2.70]	<0.001	77.60	Moderate
BAX	5	−7.89 [−10.20, −5.58]	<0.001	60.93	Moderate
BCL-2	2	5.58 [4.05, 7.12]	<0.001	0.00	High
PTEN	3	−4.91 [−6.40, −3.42]	<0.001	0.00	High
Pyroptosis					
Caspase-1	2	−2.92 [−5.65, −0.19]	0.036	74.30	Moderate
Fibrosis					
α-SMA	3	−2.01 [−2.92, −1.10]	0.004	0.00	High
Fibrosis size	3	−3.74 [−5.62, −1.86]	<0.001	0.00	High
Cell proliferation					
Ki67	2	1.92 [0.98, 2.85]	<0.001	0.00	High

Abbreviations: RIRI, renal ischemia-reperfusion injury; SCr, serum creatinine; BUN, blood urea nitrogen; Kim-1, kidney injury molecule-1; IL-1β, interleukin-1 beta; TNF-α, tumor necrosis factor-alpha; CAT, catalase; MDA, malondialdehyde; TUNEL, terminal deoxynucleotidyl transferase dUTP nick end labeling; BAX, Bcl-2-associated X protein; BCL-2, B-cell lymphoma 2; PTEN, phosphatase and tensin homolog; α-SMA, alpha-smooth muscle actin.

Subgroup analyses explored potential sources of heterogeneity. Most subgroups showed significant SCr reductions, with a marginal improvement at the 48-h estimation time point (SMD = −4.71, 95% CI: −9.36 to 0.21, *P* = 0.060) (Table 4). However, fibroblast-derived exosomes showed no significant SCr reduction *versus* controls (SMD = −0.76, 95% CI: −1.67 to 0.15, *P* = 0.101) (Table 4). Univariate meta-regression analysis demonstrated that stem cell-derived exosomes significantly reduced SCr levels compared to fibroblast-derived exosomes (Coefficient = −5.80, CI: −10.40 to −1.19, *P* = 0.014) (Table 4). Exosomes derived from endothelial colony-forming cells (ECFCs), a subtype of endothelial progenitor cells, also showed a marginally greater effect than fibroblast-derived exosomes (Coefficient = −4.61, 95% CI: −9.92 to 0.70, *P* = 0.089) (Table 4). Therapeutic efficacy was significantly higher in rats *versus* mice (Coefficient = −3.14, CI: −6.50 to −0.33, *P* = 0.030), and with ischemic durations exceeding 40 min compared to shorter durations (Coefficient = −3.68, CI: −6.71 to −0.65, *P* = 0.017) (Table 4). Additionally, pre-treatment yielded marginally greater therapeutic benefits than post-treatment (Coefficient = −4.28, CI: −9.31 to 0.75, *P* = 0.095) (Table 4).

**TABLE 4:** Subgroup analysis and univariate meta-regression of experimental variables in exosome therapy for RIRI.

Outcome	Numbers of experiments	Subgroup analysis			Meta regression	
		SMD [95% CI]	*P*	*I* ^*2*^ (%)	Coefficient [95% CI]	*P*
Animals						
Mice	8	−3.60 [−5.36, −1.83]	<0.001	88.45	Ref.	
Rats	11	−7.36 [−9.77, −4.96]	<0.001	87.16	−3.41 [−6.50, −0.33]	0.030
I/R model						
Bilateral Renal Ischemia	9	−5.52 [−7.62, −3.43]	<0.001	86.27	Ref.	
Unilateral ischemia with contralateral nephrectomy	5	−5.62 [−8.89, −2.35]	<0.001	90.93	0.03 [−4.34, 4.40]	0.990
Unilateral Renal Ischemia	5	−6.91 [−12.56, −1.27]	0.016	96.94	−0.45 [−4.98,4.08]	0.845
Exosome source						
Fibroblasts	2	−0.76 [−1.67, 0.15]	0.101	0.00	Ref.	
ECFCs	4	−5.27 [−8.05, −2.48]	<0.001	79.86	−4.61 [−9.92, 0.70]	0.089
Stem cells	13	−6.70 [−8.75, −4.65]	<0.001	89.61	−5.80 [−10.41, −1.19]	0.014
Exosome dose						
≤100 µg	12	−6.06 [−8.1, −4.02]	<0.001	88.95	Ref.	
>100 µg	7	−5.15 [−8.22, −2.07]	0.001	93.99	1.07 [−2.50, 4.65]	0.556
Administration route						
Subcapsular of the kidney	1	−4.12 [−5.86, −2.39]	<0.001	0.00	Ref.	
Intravenous	13	−5.33 [−7.31, −3.34]	<0.001	92.18	−1.28 [−8.79, 6.24]	0.739
Arterial	5	−7.25 [−11.18, −3.31]	<0.001	85.57	−3.03 [−11.06, 5.01]	0.460
Ischemic duration						
≤40 min	9	−3.61 [−2.04, −5.18]	<0.001	85.72	Ref.	
>40 min	10	−7.76 [−10.38, −5.13]	<0.001	88.15	−3.68 [−6.71, −0.65]	0.017
Administration timing						
Post-treatment	16	−5.07 [−6.67, −3.47]	<0.001	90.03	Ref.	
Pre-treatment	3	−10.53 [−18.39, −2.67]	0.009	87.41	−4.28 [−9.31, 0.75]	0.095
Estimation time point						
24 h	8	−6.40 [−9.80, −3.00]	<0.001	93.27	Ref.	
48 h	4	−4.71 [−9.63, 0.21]	0.060	96.23	1.66 [−3.27, 6.58]	0.510
72 h	5	−5.98 [−7.57, −4.39]	<0.001	50.52	−0.12 [−4.71, 4.46]	0.958
4 w	2	−5.78 [−11.04, −0.53]	0.031	83.84	0.35 [−5.97, 6.66]	0.915

Abbreviations: RIRI, renal ischemia-reperfusion injury; SMD, standardized mean difference; CI, confidence interval; I^2^, I-squared.

**FIGURE 3:**
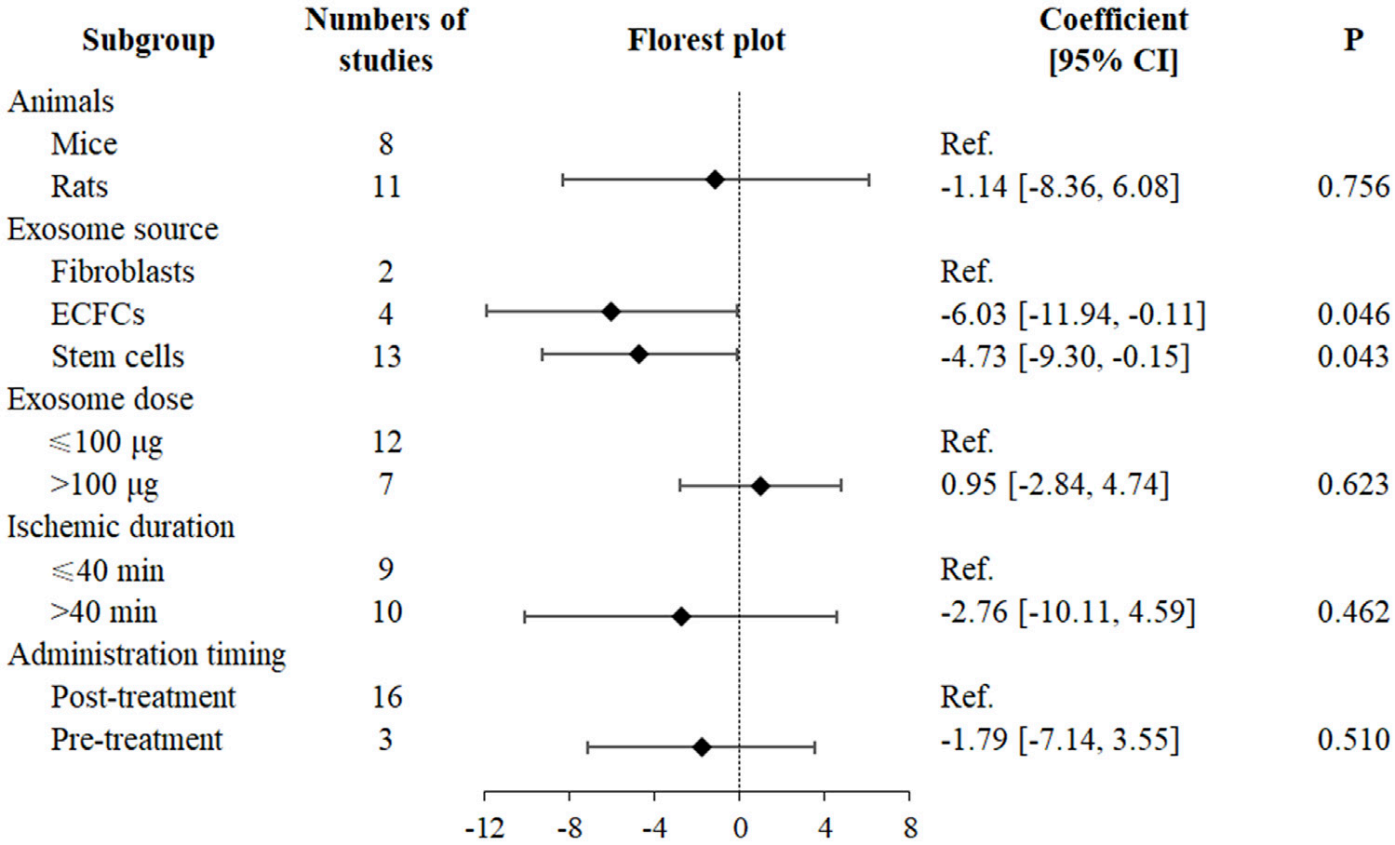
Multivariate meta-regression and forest plot in exosome therapy for RIRI. RIRI, renal ischemia-reperfusion injury; ECFCs, endothelial colony-forming cells; CI, confidence interval.

A multivariable meta-regression analysis was conducted, adjusting for animal species, exosome source, exosome dose (potentially relevant but non-significant, *P* = 0.556), ischemic duration, and administration timing (Figure 3). The analysis indicated that exosome source significantly affected SCr reduction, with ECFC-derived (SMD = −6.03, 95% CI: −11.94 to −0.11, *P* = 0.046) and stem cell-derived (SMD = −4.73, 95% CI: −9.30 to −0.15, *P* = 0.043) exosomes exhibiting greater efficacy than fibroblast-derived exosomes (Figure 3). However, no significant dose-dependent effects on SCr reduction were identified (SMD = 0.95, 95% CI: −2.84 to 4.74, *P* = 0.623) (Figure 3). Following adjustment, the effects of animal species (SMD = −1.14, 95% CI: −8.36 to 6.08, *P* = 0.756), ischemic duration (SMD = −2.76, 95% CI: −10.11 to 4.59, *P* = 0.462), and administration timing (SMD = −1.79, 95% CI: −7.14 to 3.55, *P* = 0.510) lost statistical significance (Figure 3).

**FIGURE 4:**
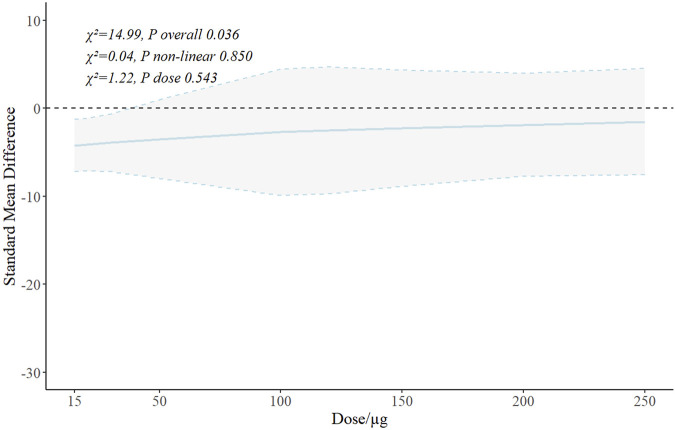
RCS curve of the relationship between exosome dose and renal function recovery. RCS, restricted cubic spline; χ^2^, chi-square.

A generalized linear model adjusted for animal species, exosome source, ischemic duration, administration timing, and study weight was applied, followed by RCS fitting (Figure 4). The Wald test indicated no nonlinear relationship between exosome dose and SCr reduction (*χ* ^*2*^ = 0.04, *P* = 0.850), and no significant effect of exosome dose on the SMD for SCr reduction (*χ* ^*2*^ = 1.22, *P* = 0.543). These findings implied the absence of a discernible dose–response relationship within the dose range investigated.

**FIGURE 5:**
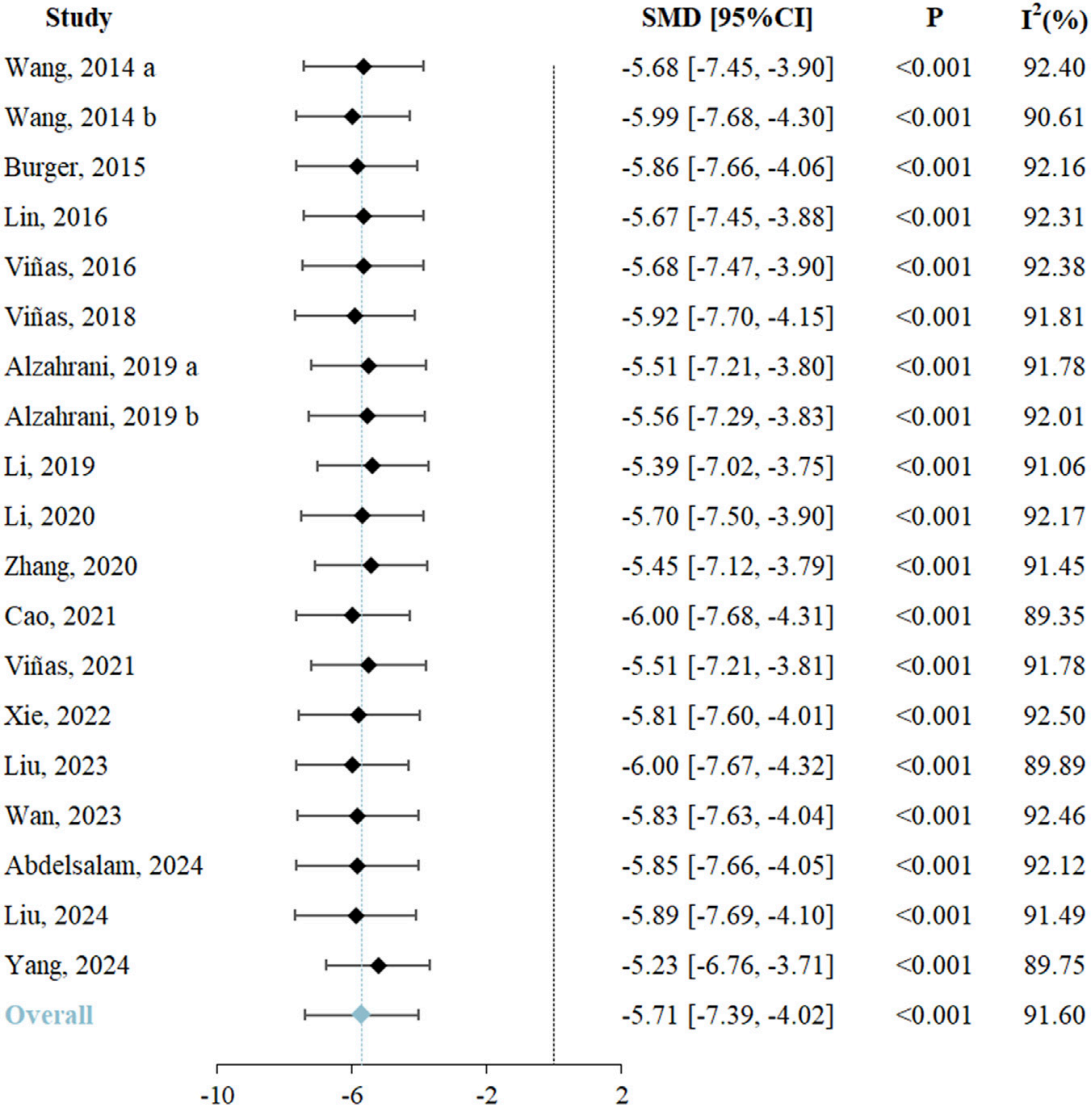
Sensitivity analysis of the exosome therapy on SCr level. SMD, standardized mean difference; CI, confidence interval; I^2^, I-squared; SCr, serum creatinine.

Sensitivity analysis confirmed that the sequential exclusion of individual studies did not substantially alter the estimated SCr reduction, supporting the robustness and consistency of the overall findings (Figure 5).

**FIGURE 6:**
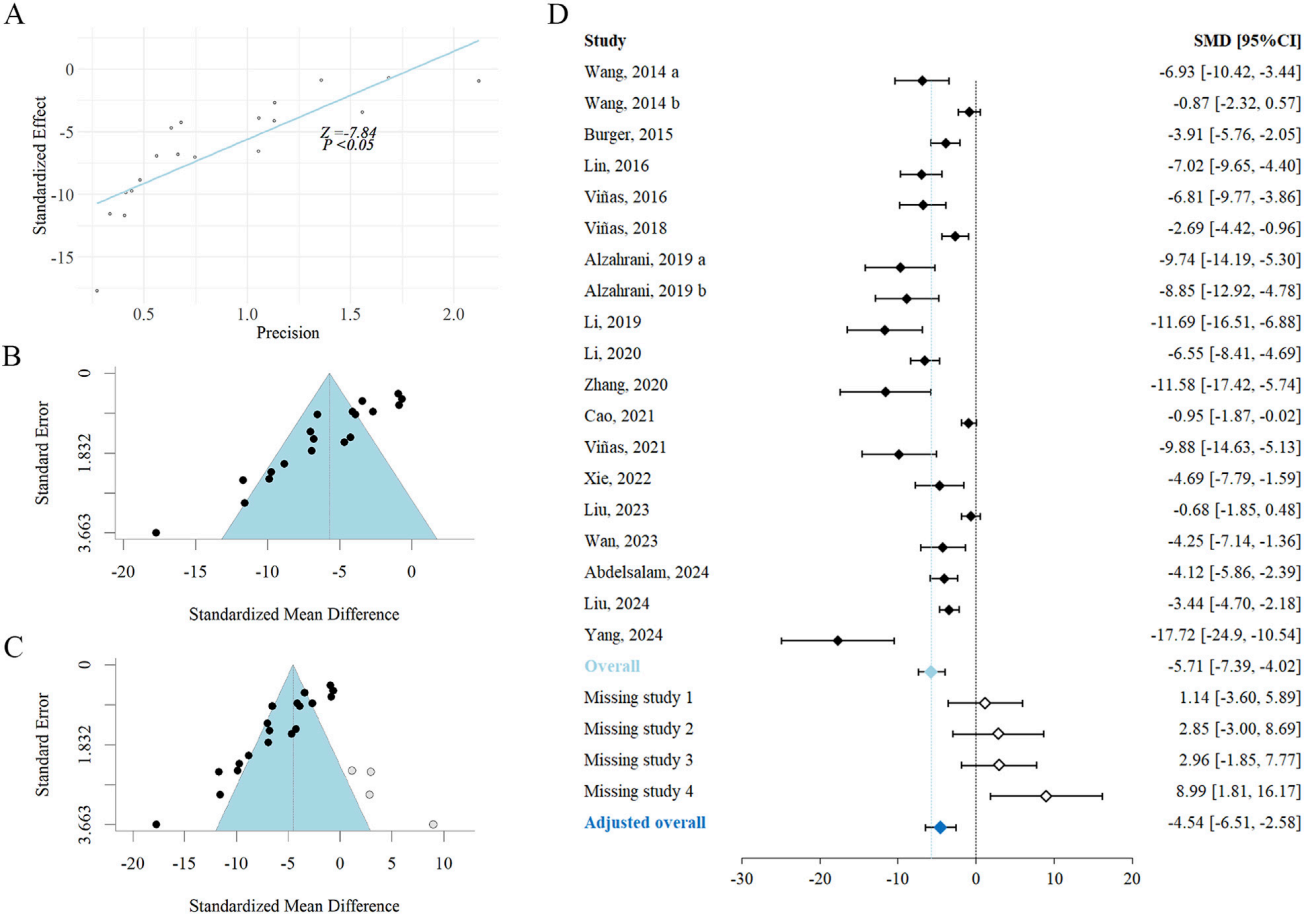
Publication bias assessment of the exosome therapy on SCr level. **(A)** Egger’s test plot, **(B)** funnel plot, and **(C)** funnel plot after trimming-and-filling for SCr. **(D)** Initial and trimming-and-filling adjusted forest plot for SCr. SMD, standardized mean difference; CI, confidence interval; SCr, serum creatinine.

Egger’s regression test suggested potential publication bias (Z = −7.84, *P* < 0.05) (Figure 6A). Funnel plot inspection revealed asymmetry, with some effect estimates falling beyond the plot boundaries, indicating a potential small-study effect (Figure 6B). To further assess potential bias, the trim-and-fill method imputed four missing studies (Figure 6C). Following adjustment, the SCr reduction remained significant (SMD = −4.54, 95% CI: −6.51 to −2.58) in exosome-treated animals, indicating that publication bias did not substantially affect the robustness of the findings (Figure 6D).

#### 3.4.2 Second outcome

##### 3.4.2.1 Renal damage

The meta-analysis revealed that exosome therapy significantly reduced BUN levels compared to controls (SMD = −4.78, 95% CI: −6.49 to −3.07, *P* < 0.001; *I* ^*2*^ = 92.22%), indicating alleviation of renal damage (Table 3; Supplementary Figure S1). Egger’s regression test suggested potential publication bias (Z = −6.67, *P* < 0.05) (Supplementary Figure S2A1). Funnel plot asymmetry prompted trim-and-fill adjustments, imputing four hypothetical missing studies (Supplementary Figure S2A2). Post-adjustment analysis confirmed sustained BUN reduction (SMD = −3.51, 95% CI: −5.58 to −1.44, *P* < 0.001), demonstrating robustness and showing no significant impact from publication bias (Supplementary Figure S2A3). The evidence level for BUN improvement was rated as moderate (Supplementary Table S3).

Exosome therapy also significantly reduced Kim-1 levels (SMD = −4.78, 95% CI: −8.01 to −3.66, *P* < 0.001; *I* ^*2*^ = 47.98%), supported by moderate level evidence (Table 3; Supplementary Figure S1).

Furthermore, exosome therapy significantly lowered kidney injury scores (SMD = −4.96, 95% CI: −6.68 to −3.23, *P* < 0.001; *I* ^*2*^ = 85.30%) (Table 3; Supplementary Figure S1). Egger’s regression test detected potential publication bias (Z = −6.01, *P* < 0.05) (Supplementary Figure S2B1). Funnel plot inspection revealed asymmetry, but subsequent trim-and-fill adjustments did not alter the result (SMD = −4.96, 95% CI: −6.68 to −3.23, *P* < 0.001), preserving the robustness of kidney injury scores reduction (Supplementary Figures S2B2 and S2B3; Supplementary Figure S2B3). The evidence level was rated as moderate (Supplementary Table S3).

##### 3.4.2.2 Inflammation

The meta-analysis demonstrated that exosome therapy did not significantly reduce IL-1β levels (SMD = −3.57, 95% CI: −8.13 to 0.99, *P* = 0.125; *I* ^*2*^ = 97.70%) (Table 3; Supplementary Figure S3). However, TNF-α levels (SMD = −3.84, 95% CI: −6.43 to −1.25, *P* = 0.004; *I* ^*2*^ = 92.92%) and neutrophil counts (SMD = −7.35, 95% CI: −9.12 to −5.58, *P* < 0.001; *I* ^*2*^ = 9.73%) were significantly reduced, indicating anti-inflammatory effects (Table 3; Supplementary Figure S3). Moderate level evidence supported reductions in IL-1β and TNF-α, while high level evidence affirmed neutrophil reduction (Supplementary Table S3).

##### 3.4.2.3 Oxidative stress

The meta-analysis demonstrated that exosome therapy significantly attenuated oxidative stress by increasing CAT levels (SMD = 12.06, 95% CI: 2.49 to 21.63, *P* = 0.014; *I* ^*2*^ = 91.39%) and decreasing MDA levels (SMD = −5.64, 95% CI: −10.34 to −0.95, *P* = 0.019; *I* ^*2*^ = 87.47%) *versus* controls (Table 3; Supplementary Figure S4). Moderate level evidence supported oxidative stress improvements (Supplementary Table S3).

##### 3.4.2.4 Apoptosis

The meta-analysis demonstrated that exosome therapy significantly suppressed apoptosis, evidenced by reduced TUNEL positive cells (SMD = −3.40, 95% CI: −4.59 to −2.22, *P* < 0.001; *I* ^*2*^ = 70.78%), decreased levels of Caspase-3 (SMD = −4.16, 95% CI: −5.61 to −2.70, *P* < 0.001; I^2^ = 77.60%), BAX (SMD = −7.89, 95% CI: −10.20 to −5.58, *P* < 0.001; *I* ^*2*^ = 60.93%), and PTEN (SMD = −4.91, 95% CI: −6.40 to −3.42, *P* < 0.001; *I* ^*2*^ = 0.00%) compared to controls (Table 3; Supplementary Figure S5). In contrast, BCL-2 levels increased (SMD = 5.58, 95% CI: 4.05 to 7.12, *P* < 0.001; *I* ^*2*^ = 0.00%) (Table 3; Supplementary Figure S5). TUNEL positive cells mark apoptotic cell death, caspase-3 acts as an executioner caspase, and BAX functions as a pro-apoptotic protein, all supported by moderate level evidence (Supplementary Table S3). PTEN, a crucial regulator of apoptosis, and BCL-2, an anti-apoptotic protein, were backed by high level evidence (Supplementary Table S3).

##### 3.4.2.5 Pyroptosis

The meta-analysis demonstrated that exosome therapy reduced caspase-1 levels (SMD = −2.92, 95% CI: −5.65 to −0.19, *P* = 0.036; *I* ^*2*^ = 74.30%), a key mediator of pyroptosis, compared to controls (Table 3; Supplementary Figure S6). Moderate level evidence supported this finding (Supplementary Table S3).

##### 3.4.2.6 Fibrosis

The meta-analysis demonstrated that exosome therapy attenuated renal fibrosis, as indicated by reduced fibrosis marker α-SMA (SMD = −2.01, 95% CI: −2.92 to −1.10, *P* = 0.004; *I* ^*2*^ = 0.00%) and decreased fibrosis size (SMD = −3.74, 95% CI: −5.62 to −1.86, *P* < 0.001; *I* ^*2*^ = 0.00%) *versus* controls (Table 3; Supplementary Figure S6). High level evidence confirmed these antifibrotic effects (Supplementary Table S3).

##### 3.4.2.7 Cell proliferation

The meta-analysis demonstrated that exosome therapy enhanced renal cell proliferation, shown by increased Ki67 expression compared to controls (SMD = 1.92, 95% CI: 0.98 to 2.85, *P* < 0.001; *I* ^*2*^ = 0.00%) (Table 3; Supplementary Figure S6). Moderate level evidence supported this finding (Supplementary Table S3).

## 4 Discussion

Exosomes, critical mediators of intercellular communication and biomolecular transport, play a crucial regulatory role in the pathophysiological mechanisms underlying renal injury and subsequent tissue repair (Ibrahim and Marbán, 2016). Their nanoscale architecture, low immunogenicity, prolonged circulation kinetics, and cargo protection capabilities make them promising biomedical agents. Emerging preclinical and clinical studies show therapeutic versatility, as evidenced by applications in cardiovascular regeneration, neuroprotection, and oncology (Wang C. et al., 2021; Zhang et al., 2023; Yu T. et al., 2024). To our knowledge, this constitutes the first systematic review and meta-analysis integrating preclinical evidence on exosomal renoprotection in RIRI, with rigorous evaluation of therapeutic outcomes across heterogeneous models. Our analysis revealed exosome therapy exerted multi-mechanistic renoprotective effects, with integrated preclinical data supporting improved renal function, alleviated kidney damage, downregulated inflammatory responses, reduced oxidative stress levels, suppressed apoptosis and pyroptosis, enhanced cellular proliferative capacity and attenuated fibrotic remodeling. These findings highlight the translational potential of exosome therapy for RIRI, particularly in preventing AKI to CKD transition, though inter-species efficacy differences require validation.

### 4.1 Therapeutic mechanism exploration

IRI initiates inflammatory cascades that induce systemic immune activation. Distinct from pathogen-induced infections, sterile inflammation in IRI originates from endogenous danger signals that trigger proinflammatory cascades post-ischemia (Chen and Nuñez, 2010). Following ischemic insult, neutrophil and monocyte infiltration into peritubular capillaries worsens microvascular congestion and impairs tissue oxygenation (Nourshargh and Alon, 2014). Activated leukocytes and injured endothelial cells release substantial reactive oxygen species (ROS) and proteases, propagating cellular damage to adjacent tissues. Subsequent cell death amplifies innate and adaptive immune responses, creating inflammation-driven pathological loops (Iyer et al., 2009; Wang W. et al., 2024). Therefore, intercepting proinflammatory signaling constitutes a key therapeutic strategy against IRI. Our meta-analysis revealed exosome therapy significantly reduced TNF-α levels and inhibits neutrophil infiltration, demonstrating potent immunomodulatory effects. Although previous studies reported IL-1β suppression, our analysis found non-significant modulation, possibly attributable to heterogeneity in exosomal sources across studies. Notably, fibroblast-derived exosomes prevalent in analyzed studies showed reduced efficacy in IL-1β regulation. The reperfusion phase triggers oxidative burst through sudden oxygen reintroduction, causing ROS-mediated damage to biomolecules such as lipids, proteins, and DNA, and consequent cellular dysfunction (Schieber and Chandel, 2014). Experimental evidence indicates exosomal activation of nuclear factor erythroid 2-related factor 2 (Nrf2) upregulates antioxidant enzymes, strengthening cellular defenses against oxidative stress (Che et al., 2024). These findings concord with our meta-analytic evidence of exosome-induced CAT elevation and membrane protection.

Severe oxidative stress in RIRI induces mitochondrial swelling and outer membrane permeabilization, releasing cytochrome C, succinate, and N-formyl peptides into cytosolic and extracellular compartments (Ye et al., 2023). This cascade activates caspase-dependent apoptosis, accelerating programmed cell death. Additionally, mounting evidence implicates caspase-1-mediated pyroptosis as another critical mechanism in IRI pathogenesis (Kolachala et al., 2021; Jin et al., 2024). Differing from apoptosis, pyroptosis features membrane permeabilization and cytosolic content release, amplifying immunostimulatory and proinflammatory cascades (Zheng et al., 2022). This self-perpetuating inflammatory cascade exacerbates tissue damage and renal dysfunction. Importantly, current therapies fail to adequately address the dual contributions of apoptosis and pyroptosis to RIRI pathology. Exosomes demonstrate dual inhibition of apoptotic and pyroptotic pathways in RIRI models, potentially enabling novel therapeutic strategies against AKI progression and chronic renal deterioration.

Emerging data suggest exosomes facilitate cellular repair and promote renal regeneration. I/R induced AKI manifests tubular epithelial cell loss, with impaired dedifferentiation and proliferative capacity obstructing renal repair (Bonventre, 2003). In a porcine AKI model, Huang et al. reported that mesenchymal stem cell (MSC)-derived exosomes enhance tubular repair *via* stimulated proliferation (Huang et al., 2022). Our meta-analysis confirmed exosome therapy consistently upregulated proliferation markers, substantiating their regenerative role in post-RIRI recovery.

Renal fibrosis, a prevalent sequela of AKI, originates from pathological interactions among dysfunctional tubules, activated fibroblasts, and immune cells within inflammatory niches (Huang et al., 2023). Our analysis indicatesd exosome therapy suppresses α-SMA expression and fibrotic lesion development, suggesting extracellular matrix remodeling pathway modulation mediates antifibrotic effects.

### 4.2 Application strategy optimization

Despite clinical use of MSCs to improve transplant outcomes, intravenous administration, whether autologous or allogeneic, commonly causes pulmonary sequestration, drastically reducing cell viability and target tissue engraftment (Li et al., 2020b; Wen et al., 2024). Functioning as potent cellular surrogates, exosomes demonstrate enhanced tissue homing capabilities. In ischemic kidneys, exosomal adhesion receptors, such as very late antigen-4 (VLA-4) and lymphocyte function-associated antigen-1 (LFA-1), selectively engage upregulated vascular cell adhesion molecule-1 (VCAM-1) and intercellular adhesion molecule-1 (ICAM-1) ligands in injured renal tissues (Ding et al., 2024). This molecular specificity drives administration route-independent therapeutic effects, evidenced by comparable functional restoration across intravenous, intra-arterial, and subcapsular delivery in our study.

High-throughput analyses reveal marked exosomal cargo heterogeneity across cellular sources and pathophysiological conditions, spanning proteomic, lipidomic, and transcriptomic dimensions (Jeppesen et al., 2019). Stem/progenitor cell-derived exosomes dominate current therapeutic pipelines due to their dual immunomodulatory and pro-regenerative capacities (Tan et al., 2024). However, accumulating evidence indicates exosome functionality exhibits source-specific specialization (Li M. et al., 2020). Han et al. reported that fibroblast-derived exosomes promoted bone-tendon healing *via* upregulation of aggrecan (ACAN), collagen type I alpha 1 (COL1A1), and COL3A1 expression, thereby promoting dense collagen fiber formation (Han et al., 2025). Notably, the disease-selective efficacy of exosomes highlights their functional specificity, as exosomes from homeostatic fibroblasts exhibit limited efficacy in early-stage RIRI, a condition primarily characterized by oxidative stress, inflammation, and tubular necrosis. This contrast in therapeutic outcomes reflects distinct functional requirements, with acute oxidative damage dominating early-stage RIRI, while chronic extracellular matrix (ECM) remodeling drives musculoskeletal repair (Yu W. et al., 2024). Furthermore, Liu et al. demonstrated that ischemic preconditioning endowed fibroblast-derived exosomes with anti-apoptotic properties *via* BCL-2 upregulation, eventually suppressing tubular cell death (Liu and Shen, 2023). This observation suggests a causal link between parental cell state transitions, from physiological adaptation to pathological priming, and functional exosome reprogramming. Consistent findings have been reported in the tumor microenvironment, wherein fibroblast-derived exosomes promote cancer cell invasion, modulate immune evasion, and induce angiogenesis, whereas these functions are generally absent in homeostatic fibroblast-derived exosomes (Chen B. et al., 2021; Li et al., 2021a; Peng et al., 2023).

Beyond mammalian exosome systems, plant-derived exosomes are emerging as xenobiotic therapeutic platforms with unique biosafety and scale-up advantages, attributable to their low immunogenicity and agricultural scalability (Dhar et al., 2024; Zhao et al., 2024). Exosomes from *Panax notoginseng* and *Momordica charantia* exhibit cross-species neuroprotection, with demonstrated capacity to attenuate cerebral IRI through PI3K/Akt pathway activation, which is a conserved cytoprotective mechanism transferable to renal and other ischemia models (Cai et al., 2022; Li et al., 2023). The absence of mammalian viral contamination risk and compatibility with industrial agricultural practices position plant-derived exosomes as a translationally superior alternative to mammalian counterparts, particularly for high-demand therapeutic applications requiring cost-effective biomanufacturing.

Current literature lacks systematic investigations into the dose-response relationship underlying exosome-mediated therapeutic effects. Our meta-analysis incorporated studies administering exosome doses of 15–250 µg. Notably, no statistically significant dose-dependent effects on exosome-mediated renoprotection were observed across this dosage spectrum. Moreover, RCS analysis revealed a paradoxical dose-dependent inversion, where higher exosome doses were associated with reduced therapeutic efficacy, though this trend was not statistically significant. This finding contradicts established preclinical evidence demonstrating positive dose-dependent therapeutic efficacy in animal models (Stenqvist et al., 2013; Xiao et al., 2025; Zhang et al., 2022). However, extant preclinical investigations are frequently constrained by critical limitations of inadequate dose intervals and restricted experimental cohorts, all of which compromise dose-response characterization. Emerging evidence indicates the existence of therapeutic windows. Zhang et al. established optimized neuroprotection with 100 µg exosomes in traumatic brain injury models, demonstrating significant superiority over both lower (50 µg) and higher (200 µg) doses (Zhang et al., 2020b). Similarly, Zhao et al. documented a nonlinear relationship between exosome concentration and neural regeneration, peaking at 0.9 × 10^10^ particles/mL through upregulation of regenerative factors. At maximal experimental concentration (7.4 × 10^10^ particles/mL), this regenerative capacity was abolished, potentially due to microvesicle-mediated neuritogenesis suppression *via* alternative signaling pathways (Zhao et al., 2020). Additionally, Saffari et al. identified a threshold effect, with 5% exosome purity maximizing neurite outgrowth (Saffari et al., 2023). It is currently widely recognized that exosomal renoprotection predominantly arises from bioactive cargo components, including proteins and nucleic acids (Simpson et al., 2009; Li et al., 2021b). Proteins are essential for cell adhesion, membrane fusion, signal transduction, and metabolic regulation, whereas miRNAs govern exosome-mediated regulatory networks. Exosome bioactivity relies on interactions with recipient cells through membrane fusion, endocytosis, or receptor-ligand binding (Gurung et al., 2021). Of particular significance, receptor-ligand binding, essential for immunomodulation and apoptosis regulation, involves exosomal transmembrane proteins engaging target cell receptors (Mathieu et al., 2019). This uptake mechanism parallels saturable drug transport kinetics, suggesting the existence of definitive absorption ceilings. Critically, supraphysiological doses lead to nonspecific binding through membrane fusion and endocytosis, perturbing signaling cascades and paradoxically diminishing therapeutic outcomes (Gurung et al., 2021; Zech et al., 2012). Additionally, excessive miRNA concentrations precipitate cytotoxicity and disrupt cellular homeostasis (Shah et al., 2016). Together, these mechanisms may partly account for the absence of a dose-dependent effect observed in our study. Collectively, these findings underscore the imperative need for precise dose optimization to enable clinical translation. Moving forward, methodologically rigorous studies are required to elucidate pathologically specific dose-response relationships, thereby delineating therapeutic optima and refining clinical safety-efficacy profiles.

Emerging evidence suggests that remote ischemic preconditioning (RIPC) facilitates multi-organ communication through exosome-mediated signaling pathways (Wang M. et al., 2024). RIPC stimulation significantly enhances exosome secretion from ischemic tissues into circulation, which is enriched in cytoprotective miRNAs such as miR-21, miR-24, and miR-126a (Minghua et al., 2018; Pan et al., 2019; Li D. et al., 2021). Notably, genetic silencing of these miRNAs completely abrogates the protective effects of RIPC-induced exosomes, confirming their essential role in ischemic preconditioning (Minghua et al., 2018). RIPC-induced exosomes are further characterized by elevated HIF-1α cargo, driving renal hypoxia adaptation through coordinating transcriptional activation of downstream effectors (Li Y. et al., 2019). These nanovesicles concomitantly deliver heat shock protein 70 (HSP70), a molecular chaperone, establishing preconditioning defenses against impending ischemic stress (Cheng et al., 2023). Furthermore, the RIPC procedure stimulates exosome-mediated nicotinamide adenine dinucleotide phosphate (NADPH) oxidase transfer, generating a controlled ROS burst that activates PI3K/Akt and Nrf2 signaling cascades, thereby potentiating tissue resistance (Benavides et al., 2023).

Exosomal pre-treatment mirrors the protective strategy of RIPC, with stem/progenitor cell-derived exosomes functioning as multimodal therapeutic agents targeting a broad spectrum of pathogenic pathways. Through the transfer of miRNAs, exosomes exert antioxidant, anti-inflammatory, and anti-apoptotic effects, thereby enhancing tissue resistance to ischemia and suppressing early inflammatory cascades (Raposo and Stoorvogel, 2013). Our univariate meta-regression analysis revealed that while both pre-treatment and post-treatment confer renoprotective effects, pre-treatment demonstrates superior functional improvement. However, this advantage may diminish after adjusting for covariates. Mechanistically, pre-treatment enables healthy renal cells to actively internalize exosomes, leading to early upregulation of stress-resistance genes and enhanced cytoprotective responses (Liu et al., 2023). In contrast, post-treatment efficacy is limited by ATP depletion and membrane damage in post-ischemic cells, restricting therapeutic engagement to surviving subpopulations (Pham et al., 2024). Therefore, early exosome administration is more effective in mitigating acute injury through antioxidant and anti-inflammatory mechanisms, whereas delayed administration primarily contributes to tissue repair and remodeling (Feng et al., 2024). This dual-phase therapeutic strategy highlights exosomes as versatile interventions for RIRI, providing both prophylactic and therapeutic benefits throughout the injury continuum.

### 4.3 Clinical translational challenges

#### 4.3.1 Study limitation

Despite comprehensive inclusion of studies meeting predefined criteria, this meta-analysis presents four principal limitations requiring critical appraisal. Firstly, translational validation remains constrained by predominant reliance on rodent models. Secondly, as research on exosome therapy for RIRI remains exploratory, the limited evidence and small sample size reduced statistical power and hindered further analysis of stem cell subtype-specific effects. Thirdly, although considerable heterogeneity among the included studies, which may have influenced the robustness of the results, was partly eliminated, the limited data volume prevented further exploration of heterogeneity sources, including differences in stem cell subtypes and variations in exosome isolation and extraction methods. Finally, the narrow investigational dose range (15–250 μg, median 100 μg) precludes definitive characterization of dose-response relationships.

#### 4.3.2 Enhancing mechanistic understanding

Exosomes derived from stem/progenitor cells have demonstrated broad therapeutic potential across various disease models (Clua-Ferré et al., 2024; Li et al., 2020d). Their efficacy is closely linked to the unique cargo they carry, particularly regulatory biomolecules such as miRNAs and functional proteins, which participate in complex cellular signaling networks (Kalluri and LeBleu, 2020). These exosomal contents can be transferred to target cells to modulate cellular activities and contribute to damage mitigation. However, as previously discussed, the functional plasticity of exosomes is highly dependent on the cellular origin and pathological stress conditions. Therefore, a deeper exploration of the genomic and proteomic profiles of exosomes from different contexts is essential for selecting the most appropriate type for optimal therapeutic outcomes (Du et al., 2023). In parallel, plant-derived exosomes have emerged as promising bioactive agents with potential therapeutic effects (Kim et al., 2022). Nonetheless, their active constituents and mechanisms of action remain to be fully elucidated. Future research should prioritize the isolation and characterization of plant-derived exosome cargo and investigate their interactions with mammalian cells in various physiological and pathological contexts. These advances may accelerate a paradigm shift towards plant-based nanotherapeutics in addressing the global burden of ischemic diseases.

#### 4.3.3 Optimizing therapeutic strategies

Integrating intrinsic targeting properties of exosomes with advanced engineering techniques establishes a powerful foundation for precision medicine. Intranasal administration enhances targeting of the central nervous system, whereas oral routes effectively target the gastrointestinal tract (Gao et al., 2022; Wang et al., 2023). Yet the route to the kidneys remains heavily reliant on intravenous administration, an old classic, reliable but rather unimaginative. Unmodified exosomes naturally accumulate in organs with abundant mononuclear phagocytic activity, including the lungs, kidneys, liver, and spleen (Choi et al., 2021). Active homing to specific pathological microenvironments can be effectively achieved by genetically introducing targeting peptides into donor cells. For example, Zhao et al. engineered exosomes by transfecting donor cells with an Arg-Gly-Asp (RGD)-Lamp2b plasmid, allowing specific interaction with integrin αVβ3 on tumor endothelial cells (Zhao et al., 2022). Alternative approaches, such as covalent incorporation of RGD-modified 1,2-distearoyl-sn-glycero-3-phosphoethanolamine–polyethylene glycol (DSPE-PEG-RGD) into the exosomal membrane, also facilitate surface presentation of RGD peptides (Al Faruque et al., 2022). Beyond oncology applications, RGD motifs selectively target injured renal tubular cells, suggesting promising therapeutic potential for RIRI (Noiri et al., 1996). Apart from targeting considerations, the optimal timing of exosome administration is critical for enhancing therapeutic outcomes. During the early phase of IRI, ROS generation and neutrophil infiltration dominate, necessitating early administration of antioxidant-rich exosomes. Conversely, the middle-to-late stages involve prolonged activation of pro-fibrotic pathways and maladaptive tissue remodeling, indicating the need for anti-fibrotic exosomal treatments (Grace, 1994). Therefore, future exosome-based therapeutic strategies for RIRI should emphasize spatial biodistribution and timing of administration to optimize therapeutic effects.

Additionally, engineered exosomes show great promise as versatile vehicles for targeted drug delivery. MiRNAs are critical functional components within exosomes, whose therapeutic potential can be effectively exploited by targeted loading (Zhang et al., 2015). Therapeutic miRNAs can be loaded *via* genetic methods like lentiviral transfection or physical approaches, including electroporation, sonication, freeze–thaw cycling, and extrusion (Ran et al., 2020; Luan et al., 2017; Wang J. et al., 2021; Chen H. et al., 2021). Notably, physical loading methods are increasingly preferred due to their higher efficiency, simplicity, and lower complexity compared to viral vectors (Tenchov et al., 2022). Combining targeting peptides and therapeutic cargoes into exosomes significantly enhances their therapeutic efficacy. For instance, Lai et al. utilized lentiviral plasmids to load miR-193b-3p and neuron-targeting rabies virus glycoprotein (RVG) peptides into MSC-derived exosomes (Lai et al., 2020). The resulting exosomes specifically accumulated in ischemic brain regions, significantly reducing neuroinflammation and mitigating early brain injury in mice following subarachnoid hemorrhage. Besides RNA molecules, engineered exosomes can efficiently load various small-molecule therapeutic drugs. Jia et al. loaded curcumin, a small molecule with anti-inflammatory and immunomodulatory properties, into neuron-targeting (Arg-Gly-Glu) RGE-modified exosomes by electroporation, effectively demonstrating therapeutic potential against glioma (Jia et al., 2018). Similar approaches have been used for encapsulating chemotherapeutic agents, such as paclitaxel and doxorubicin, underscoring the versatility of engineered exosomes as delivery platforms for diverse disease applications (Yang et al., 2015; Pascucci et al., 2014; Tian et al., 2014). Furthermore, engineered exosomes have been incorporated into hybrid systems involving hydrogels, magnetic nanoparticles, and scaffolds to enhance their stability, achieve controlled release, and improve targeted delivery capabilities (Fan et al., 2024; Li et al., 2022; Kang et al., 2022). An optimal therapeutic strategy should integrate rational exosome engineering with disease-specific delivery demands, balancing cargo loading efficiency, targeting specificity, and release kinetics to achieve maximal therapeutic benefit while minimizing off-target effects.

#### 4.3.4 Advancing clinical translation

Bridging the gap between laboratory findings and clinical application is pivotal to the successful translation of exosome-based therapies for IRI. This includes conducting clinical trials to evaluate the safety and efficacy of exosome-based interventions, as well as identifying the most effective strategies for their use across different tissues. Although exosomes have progressed from preclinical animal studies to early human investigations in recent years, no clinical trials have yet specifically focused on their application in IRI. Additionally, large animal studies and randomized controlled trials (RCTs) are needed to define dose–response relationships and therapeutic thresholds.

The translation of exosome therapies into clinical practice is hindered by the lack of standardized protocols for production, isolation, and storage. Preliminary evidence from preclinical studies highlights potential therapeutic applications, but critical challenges persist. The progression from static Petri dishes to shake flasks and scalable bioreactors has markedly enhanced production efficiency (Herrmann et al., 2021). Recent innovations, particularly hollow fiber and stirred tank bioreactors, have shifted the two-dimensional (2D) static culture model to dynamic three-dimensional (3D) systems (de Almeida Fuzeta et al., 2020; Phan et al., 2018). This transition optimizes scalable production strategies, enabling reliable exosome supply for clinical applications. Current methods for exosome isolation, such as ultrafiltration, immunoaffinity separation, polymer-based precipitation, chromatography, and microfluidics, purify exosomes based on size, density, or surface properties (Kimiz-Gebologlu and Oncel, 2022). However, these techniques remain costly, time-intensive, and limited in scalability, necessitating the development of efficient strategies for large-scale clinical applications. Traditional storage techniques, including cryopreservation and lyophilization, are being optimized with increasingly effective cryoprotectants and antifreezes to maintain exosome integrity under clinical preservation and delivery conditions (Emami et al., 2018; Zhang et al., 2020c). Recently, Jia et al. utilized zeolitic imidazolate framework-8 (ZIF-8), a metal-organic framework composed of zinc ions and 2-methylimidazole, as a protective coating for exosomes, which significantly prolonged their storage stability compared to conventional preservation methods (Jia et al., 2025). Furthermore, it is crucial to standardize these processes according to Good Manufacturing Practice (GMP) guidelines to ensure the safety, consistency, and efficacy of the final manufacture. Future efforts should concentrate on refining biomanufacturing workflows to enable scalable, reliable handling of exosomes, thereby facilitating their successful clinical application in RIRI therapy.

## 5 Conclusion

This meta-analysis established moderate-to high-level evidence for exosome-mediated renoprotection in RIRI. The findings suggest that exosome therapy improved renal function, alleviated kidney damage, downregulated inflammatory responses, reduced oxidative stress levels, suppressed apoptosis and pyroptosis, enhanced cellular proliferative capacity and attenuated fibrotic remodeling. Further rigorous analysis reveals that therapeutic efficacy was critically dependent on exosomal source. Additionally, an expected positive dose-dependent relationship was not observed, suggesting the presence of an optimal therapeutic dosage window. Administration routes demonstrated no significant efficacy variance. However, the advantage of pre-treatment compared to post-treatment diminished after adjusting for covariates, indicating that temporal therapeutic windows require rigorous characterization. Subsequent investigations should prioritize exosome application to maximize therapeutic potential and expedite clinical translation for RIRI management.

## Data Availability

The original contributions presented in the study are included in the article/Supplementary Material, further inquiries can be directed to the corresponding authors.

## Glossary

RIRI: renal ischemia-reperfusion injury
AKI: acute kidney injury
SMDs: standardized mean differences
IRI: ischemia-reperfusion injury
MVBs: multivesicular bodies
PRISMA: Preferred Reporting Items for Systematic Reviews and Meta-Analyses
I/R: ischemia/reperfusion
PBS: phosphate-buffered saline
SCr: serum creatinine
BUN: blood urea nitrogen
Kim-1: kidney injury molecule-1
IL-1β: interleukin-1 beta
TNF-α: tumor necrosis factor-alpha
CAT: catalase
MDA: malondialdehyde
TUNEL: terminal deoxynucleotidyl transferase dUTP nick end labeling
BAX: Bcl-2-associated X protein
BCL-2: B-cell lymphoma 2
PTEN: phosphatase and tensin homolog
α-SMA: alpha-smooth muscle actin
SYRCLE: Systematic Review Center for Laboratory Animal Experimentation
GRADE: Grading of Recommendations Assessment Development and Evaluation
CI: confidence interval
**χ** ^**2**^: chi-square
**I** ^**2**^: I-squared
**τ** ^**2**^: tau-squared
RCS: restricted cubic spline
ECFC: endothelial colony-forming cell
ROS: reactive oxygen species
Nrf2: nuclear factor erythroid 2-related factor 2
MSC: mesenchymal stem cell
VLA-4: very late antigen-4
LFA-1: lymphocyte function-associated antigen-1
VCAM-1: vascular cell adhesion molecule-1
ICAM-1: intercellular adhesion molecule-1
ACAN: aggrecan
COL1A1: collagen type I alpha 1
RIPC: remote ischemic preconditioning
HSP70: heat shock protein 70
NADPH: nicotinamide adenine dinucleotide phosphate
RGD: Arg-Gly-Asp
DSPE-PEG-RGD: RGD-modified 1,2-distearoyl-sn-glycero-3-phosphoethanolamine-polyethylene glycol
RVG: rabies virus glycoprotein
RCTs: randomized controlled trials
2D: two-dimensional
3D: three-dimensional
ZIF-8: zeolitic imidazolate framework-8
GMP: Good Manufacturing Practice
